# Hypermethylated CDO1 and CELF4 in cytological specimens as triage strategy biomarkers in endometrial malignant lesions

**DOI:** 10.3389/fonc.2023.1289366

**Published:** 2023-12-01

**Authors:** Bingli Qi, Ye Sun, Yaohua Lv, Pei Hu, Yanli Ma, Wenying Gao, Shumei Li, Xin Zhang, Xitong Jin, Yuligh Liou, Pei Liu, Shikai Liu

**Affiliations:** ^1^ Department of Gynecologic Oncology and Surgery, Cangzhou Central Hospital, Cangzhou, Hebei, China; ^2^ Department of Pharmacy, Cangzhou Central Hospital, Cangzhou, Hebei, China; ^3^ Department of Medical Laboratory, Beijing Origin-Poly Bio-Tec Co., Ltd., Beijing, China

**Keywords:** abnormal uterine bleeding, postmenopausal bleeding, atypical hyperplasia, endometrial cancer, diagnosis, DNA methylation biomarker, cysteine dioxygenase type 1 (CDO1), UGBP Elav-like family member 4 (CELF4)

## Abstract

**Objective:**

Developing a non-invasive and reliable triage test for endometrial malignant lesions is an important goal, as it could help to reduce the number of invasive diagnostic procedures required and improve patient survival. We aimed to estimate the diagnostic value of DNA methylation levels in cervical cytological samples of endometrial cancer (EC) and endometrial atypical hyperplasia (AH).

**Methods:**

A total of 607 women who had indications for endometrial biopsy in the Department of Obstetrics and Gynecology of Cangzhou Central Hospital from October 2022 to April 2023 were enrolled in this study. The cervical exfoliated cells were collected for gene methylation before endometrial biopsy. Clinical information, tumor biomarkers, and endometrial thickness (ET) of transvaginal ultrasonography (TVS) were also collected. With endometrial histopathology as the gold standard, multivariate unconditional logistic regression was applied to analyze the risk factors of endometrial malignant lesions. The role of cysteine dioxygenase type 1 (*CDO1*) and CUGBP Elav-like family member 4 (*CELF4*) gene methylation as a triage strategy biomarker in endometrial malignant lesions was specifically explored.

**Results:**

Multivariate logistic regression analysis showed that premenopausal ET ≥ 11 mm or postmenopausal ET ≥ 5 mm, *CDO1* ΔCt ≤ 8.4, or *CELF4* ΔCt ≤ 8.8 were the risk factors for AH and EC, with odds ratios (ORs) (95%CI) of 5.03 (1.83–13.82) and 6.92 (1.10–43.44), respectively (p-values < 0.05). The sensitivity and specificity of *CDO1/CELF4* dual-gene methylation assay for AH and EC reached 84.9% (95%CI: 75.3%–94.5%) and 86.6% (95%CI: 83.8%–89.5%), respectively. ET combined with DNA methylation detection further improved the specificity to (94.9%, 95%CI: 93.1%–96.8%).

**Conclusion:**

The accuracy of cervical cytology DNA methylation is superior to that of other clinical indicators in the non-invasive examination of endometrial malignant lesions. DNA methylation combined with TVS can further improve the specificity and is a promising biomarker triage strategy in women with suspected endometrial lesions.

## Introduction

Endometrial cancer (EC) is the most prevalent gynecological cancer in countries with high income, and its occurrence is increasing worldwide (1–3). In 2017, the worldwide incidence and mortality rates of EC were 35.7 and 5.3 per 100,000, respectively. As reported by the National Cancer Center in 2019, the incidence and mortality rates of EC were respectively 10.28 and 1.9 per 100,000 in China, and both rates are increasing (4). Approximately 70% of EC patients are diagnosed in the early clinical stage and have a good prognosis because they are confined to the uterus. The prognosis of EC is influenced by factors such as age, stage, tumor differentiation, and pathological type. Patients with advanced age, late stage, and low differentiation tend to have a worse prognosis (5, 6). There is a significant prognostic difference between the histological types of endometrial cancers. Most ECs are well to moderately differentiated and develop in the presence of endometrial hyperplasia. These tumors are also known as type I (low-grade) endometrial carcinomas, as the main pathological type (7). They are associated with long-duration unopposed estrogenic stimulation. Early detection and treatment can result in a 5-year survival rate greater than 80% (8). Type II tumors are non-hormone-dependent, mainly including serous carcinoma, clear cell carcinoma, and carcinosarcoma, which are highly graded and have a tendency to recur even at an early stage, accounting for 70% of EC deaths (9). The chance of developing endometrial hyperplasia without atypia into EC is approximately 3%, but the chance increases up to 23% in developing into endometrial atypical hyperplasia (AH) (10). It is crucial to screen for AH and early EC in women at high risk (11).

Risk factors for endometrial cancer include high body mass index (BMI) (12, 13), metabolic syndrome (14, 15), diabetes mellitus (16), nulliparity and infertility, and polycystic ovarian syndrome (PCOS) (17, 18). Additional factors that increase the risk of EC include unopposed estrogen therapy, estrogen-producing tumors, and early menarche or late menopause (19). Abnormal uterine bleeding (AUB) (20) and postmenopausal bleeding (PMB) (21) are common clinical symptoms of EC. As part of routine health surveillance, clinicians should ask patients about postmenopausal and abnormal bleeding (22). Annual transvaginal ultrasound (TVS) is recommended for monitoring endometrial thickness in those with the above-mentioned factors of increased risk of endometrial cancer. Screening for endometrial cancer is recommended if ultrasonography reveals endometrial thickness (ET) >11 mm in the premenopausal (>5 mm in postmenopausal) (23) or increased blood vessels, uneven endometrium, or poor sound transmission of uterine cavity effusion (13).

There is currently a lack of well-established and universally accepted standardized screening methods for EC that exhibit both high sensitivity and standardization (24). TVS is a non-invasive diagnostic test, but the cutoff value for endometrial thickness remains uncertain, although TVS has a high sensitivity but a low specificity of 24.3%–74.0%. As a result, it has a low positive predictive value and a high rate of false-positive results, making it unreliable for identifying malignant lesions (25, 26). Traditional dilation and curettage are relatively effective methods, and hysteroscopic biopsy can be conducted to obtain a definite diagnosis, which is considered the most reliable for diagnosing EC (27). Biopsy is a highly accurate method (with a high sensitivity of 90%–100% and a specificity of 98%–100%) for detecting EC. Hysteroscopic localization biopsy or diagnostic curettage may be considered for patients at high risk of EC or with ultrasound abnormalities, but these invasive procedures to obtain endometrial tissue are also usually painful and frightening for the patients (28, 29).

Early detection is crucial for improving outcomes and reducing mortality rates, and therefore, researchers are actively investigating biomarkers that can be used for early screening and detection. Recent studies on epigenetics have shown a strong connection between DNA methylation and the progression of cancer (30). Advances in understanding the mechanisms of tumorigenesis have also led to the identification of biomarkers that can be used for early detection, diagnosis, and prognosis of cancer patients (31). Some of these methylation biomarkers have been successfully used to differentiate cancerous tissue from normal tissue with a high accuracy rate of over 95% in common cancers (32). Furthermore, there are noticeable differences in methylation levels between tissue specimens of EC and precancerous lesions compared to benign endometrium (BE) (33, 34). DNA methylation markers can be utilized to assess women with abnormal vaginal bleeding in order to differentiate between those women with endometrial carcinoma from the majority of women without malignancy (35). Recent studies have indicated that endometrial lesions can be detected through cervical cytology samples. Reijene C et al. showed that cervical cytology is a less invasive method that yields comparable results to histology in terms of diagnosis performance (with a sensitivity of 78% and a specificity of 97%). Even with cervicovaginal self-sampling, a significant number of EC cases can be diagnosed (with a sensitivity of 67% and a specificity of 97%) (36). In summary, the use of DNA methylation testing in cervical cytology samples shows promise for the diagnosis of EC and AH. Two hypermethylated candidate genes, namely, cysteine dioxygenase type 1 (*CDO1*) and CUGBP Elav-like family member 4 (*CELF4*), have been identified as potential biomarkers for endometrial cancer (33, 37–39). As a key enzyme in cysteine catabolism, CDO1 belongs to the non-heme Fe(II) dioxygenase family. CDO1 is involved in a spectrum of physiological processes, including lipid metabolism and adipogenesis (40), osteoblast differentiation (41), regulation of redox homeostasis, fertility (42), bile acid metabolism, sulfide metabolism, and growth. Many of these processes are regulated directly or indirectly by the CDO1-mediated metabolism of cysteine (43). The degree of methylation of the *CDO1* promoter is closely related to tumor progression and malignancy, and overexpression of *CDO1* promotes ferroptosis in cancer cells (43). CELF4 is involved in both co-transcriptional and post-transcriptional RNA processing. Although CELF4 proteins all appear to affect pre-mRNA splicing, they play different roles in regulating mRNA stability and translation (44). *CELF4* was a prognostic factor for EC patients; when combined with other clinical factors, the expression level of *CELF4* could effectively predict the prognosis of EC patients (38).

In the present study, we aim to propose a promising method for detecting early endometrial malignant lesions. The sensitivity and specificity of the two gene assays of *CDO1* and *CELF4* were examined on endometrial pathological results. The area under the curve (AUC) of methylation assay receiver operating characteristic (ROC) was also detected to estimate the diagnostic value for EC and AH. Finally, its methylation performance was combined with TVS to explore the possibility of multiple scenarios of methylation detection.

## Methods

### Study design and sample collection

This prospective cross-sectional study uses a non-invasive clinical method to verify the clinical efficacy of *CDO1* and *CELF4* gene methylation detection. A total of 607 women with indications for endometrial biopsy in the Department of Obstetrics and Gynecology of Cangzhou Central Hospital from October 2022 to April 2023 were enrolled in this study. Indications for endometrial biopsy include irregular vaginal hemorrhage or blood secretions after menopause or before menopause for non-cervical lesions, patients with no ovulation infertility for many years, persistent vaginal discharge, and endometrial abnormal thickening or uterine cavity mass (45–47). Collected patient clinical information and cervical exfoliated cells were collected before surgery for DNA methylation detection. The study was approved by the Institutional Review Board (IRB) of the Ethics Committee, Cangzhou Central Hospital, Hebei, China [No. 2023-144-02(z)]. Based on the Chinese Guidelines for Menopause Management and Menopausal Stimulant Therapy (48), the menopausal status of patients was determined according to clinical manifestations and examinations. Patients with indications for endometrial biopsy were enrolled, and they provided signed informed consent according to the Standards for Reporting of Diagnostic Accuracy Studies (STARD) in clinics (49). Exclusion criteria were as follows: women with a previous diagnosis of cancer in any organ, who had previously undergone hysterectomy, who did not complete all the examinations, who were receiving hormonal therapy for menopausal symptoms in 1 year, or who were receiving immunosuppressive therapy 1 year before enrollment were excluded.

### Demographic characteristics and clinical assessment

The general information and clinical manifestations of patients related to the onset of endometrial malignant lesions were collected by a special case collection table. It includes age, menopausal status, past medical history, BMI, endometrial thickness assessed by TVS within 1 month before hysteroscopy, and carbohydrate antigen 125 (CA125) value within 1 month before hysteroscopy. It also includes the history of gynecological diseases, with special attention to uterine, ovarian, and vaginal diseases. For EC patients, the EC tissue type, International Federation of Gynecology and Obstetrics (FIGO) grade and stage, and tumor history were also recorded. BMI was calculated as weight in kilograms divided by height in meters squared. The level of CA125 in serum was detected using a CA125 ELISA kit (Coibo Bio Co., Shanghai, China) according to the manufacturer’s instructions. ET was measured by TVS. In this study, the positive findings of TVS evaluation were defined as endometrial thickness ≥ 11 mm (premenopausal) and endometrial thickness ≥ 5 mm (postmenopausal). BMI ≥ 25 kg/m^2^, CA125 ≥ 35 U/ml is defined as abnormal.

### *CDO1* and *CELF4* hypermethylation detection

Methylation detection was performed in a certified DNA laboratory, and the operators and staff members were blinded to the patient’s clinical information, TVS, hysteroscopy, and histopathology results. *CDO1* and *CELF4* methylation detection (CISENDO^®^) used the specimens collected in the PreservCyt^®^ solution. Genomic DNA (gDNA) was extracted from the exfoliated cervical cell sample using the JH-DNA Isolation and Purifying kit (OriginPoly Bio-Tec Co., Ltd., Beijing, China) per the manufacturer’s instructions. The DNA concentration was quantified using the NanoDrop 2000c spectrophotometer (Thermo Fisher Scientific, Wilmington, DE, USA). Briefly, 500 ng of gDNA was subjected to bisulfite conversion using JH-DNA Methylation-Lightning MagPrep (OriginPoly Bio-Tec Co., Ltd., Beijing, China). Subsequently, the levels of *CDO1^m^* and *CELF4^m^* were determined using the CISENDO DNA Methylation Detection Kit for Endometrial Cancer (real-time PCR) with glyceraldehyde-3-phosphate dehydrogenase (*GAPDH*) as an internal control (OriginPoly Bio-Tec Co., Ltd., Beijing, China) using the ABI 7500 real-time PCR System platform (Life Technologies, Foster City, CA, USA) per the manufacturer’s instructions. The hypermethylation level of the *COD1* gene was determined by the difference between the two Ct values (ΔCt *CDO1* = Ct *CDO1* − Ct *GAPDH* and ΔCt *CELF4* = Ct *CELF4* − Ct *GAPDH*). A positive result of the CISENDO methylation (CISENDO^®^) test is defined as either *CDO1^m^* (+): ΔCt *CDO1* ≤ 8.4 or *CELF4^m^* (+): ΔCt *CELF4* ≤ 8.8.

### Statistical analysis

SPSS 27.0 (IBM Corp., Armonk, NY, USA) and R (version 4.1.2, Vienna, Austria) were used for all statistical analyses. The participants were characterized using descriptive statistics, and patients and tumor characteristics were tabulated. Use case (%) of counting data was used for comparison between groups χ^2^ test. The normality test was used to determine whether the variance of the population was equal to the Kolmogorov–Smirnov test, the measurement data of non-normal distribution was expressed by M (Q1, Q3), and the comparison between the two groups adopts the non-parametric Mann–Whitney U test. A multivariate logistic regression model was used to analyze the related factors of AH and EC. ROC curves were used to evaluate the AUCs of ET, *CDO1^m^*, or *CELF4* ^m^. Sensitivity, specificity, positive predictive value (PPV), and negative predictive value (NPV) for detecting AH and EC and their 95% confidence intervals were calculated. The methylation cutoff value for the final clinical statistical analysis was based on the CISENDO methylation (CISENDO^®^) test defined as either ΔCt *CDO1* ≤ 8.4 or ΔCt *CELF4* ≤ 8.8. All differences were considered two-sided and statistically significant at p < 0.05.

## Results

### Participant characteristics

**Figure 1:**
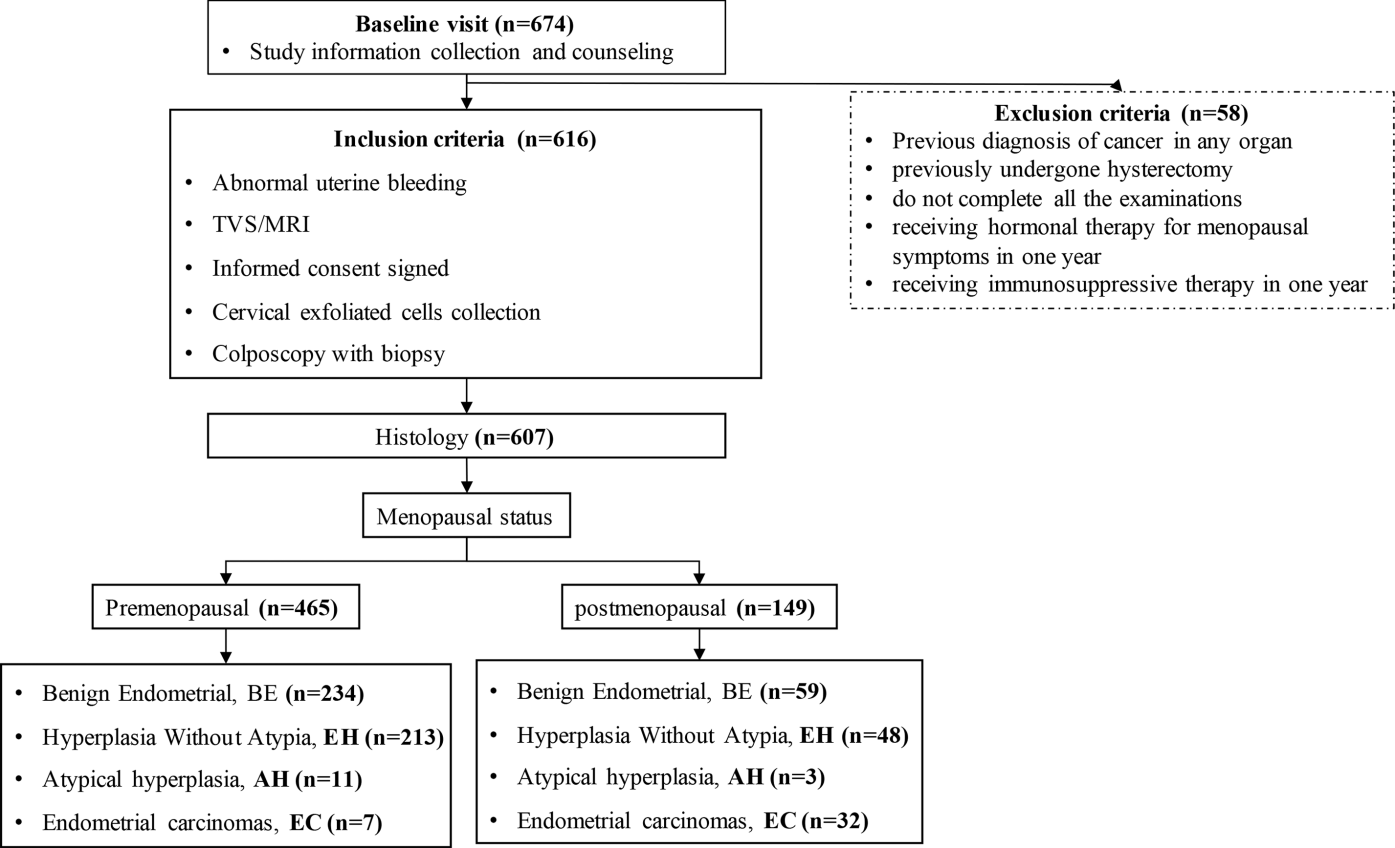
STARD diagram showing the flow of participants in the study. STARD, Standards for Reporting of Diagnostic Accuracy Studies.

**Figure 2:**
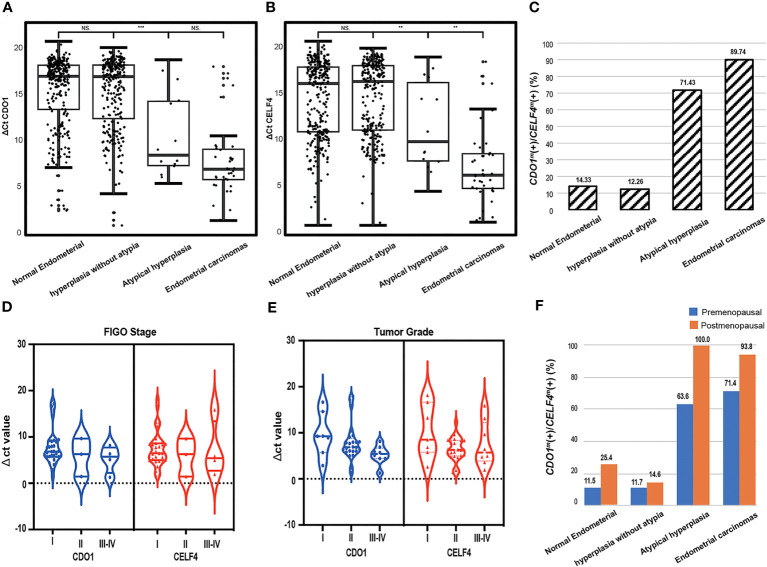
The dot plots show the distribution of DNA methylation in different degrees of pathological groups; the median and interquartile ranges are depicted by boxes. **(A)** Distribution of the value of methylated *CDO1* in different degrees of pathological results. **(B)** Distribution of the value of methylated *CELF4* in different degrees of pathological results. **(C)** The positive rate of *CDO1^m^* (+)/*CELF4* ^m^(+) in different groups of pathological results, *CDO1^m^* (+)/*CELF4* ^m^(+): ΔCt *CDO1* ≤ 8.4 or ΔCt *CELF4* ≤ 8.8. **(D)** Distribution of the value of methylated *CDO1* and *CELF4* in different FIGO stages. **(E)** Distribution of the value of methylated *CDO1* and *CELF4* in different tumor grades. **(F)** The positive rate of *CDO1^m^* (+)/*CELF4* ^m^(+) in different degrees of pathological results with different menopausal states. FIGO, International Federation of Gynecology and Obstetrics. NS, no statistical significance; **p < 0.01, ***p < 0.001.

The enrollment flowcharts and the baseline characteristics are shown in **Figure 1**; **Supplementary S1**. A total of 607 women (median age [range], 47.0 [40.0–53.0] years) were enrolled and analyzed in the study: 293 (48.3%) with benign endometrial (BE) (48.0 [42.0–52.0] years), 261 (49.2%) with hyperplasia without atypia (EH) (45.0 [38.0–51.0] years), 14 (2.3%) with AH (48.5 [42.0–54.0] years), and 39 (6.0%) with EC (56.0 [54.5–64.5] years). There is a significant difference in age between each group (p < 0.05, **Table 1**). In **Figure 2A**, there was a significant difference in ΔCt *CDO1* value between EH with AH (p < 0.05), but there were no significant differences in the remaining adjacent subgroups. There was a significant difference at ΔCt *CELF4* value between EH with AH, and AH with EC (p < 0.05, **Figure 2B**). The positivity rate of *CDO1^m^*/*CELF4^m^* in AH (71.43%) and EC (89.74%) was significantly higher than that in the BE (14.33%) and EH (12.26%) groups (p < 0.05, **Figure 2C**).

**Table 1:** The general information of different pathological groups.

	BE (n = 293)	EH (n = 261)	AH (n = 14)	EC (n = 39)	p-Value
**Age (median [IQR])**	48.00 [42.00, 52.00]	45.00 [38.00, 51.00]	48.50 [42.00, 54.00]	56.00 [54.50, 64.50]	<0.001
**Endometrial thickness (median [IQR])**	7.50 [5.00, 10.00]	9.00 [7.00, 11.11]	10.55 [8.50, 13.50]	11.11 [10.00, 15.00]	<0.001
**CA125 (median [IQR])**	18.35 [12.00, 38.10]	16.16 [10.47, 29.32]	18.90 [10.80, 21.70]	19.40 [10.90, 27.10]	0.444
**BMI (median [IQR])**	24.44 [22.48, 27.18]	24.46 [22.27, 27.34]	25.98 [22.89, 27.83]	27.18 [22.31, 30.86]	0.1
**ΔCt *CDO1* (median [IQR])**	16.86 [13.29, 18.06]	16.83 [12.30, 18.08]	8.34 [7.21, 14.16]	6.82 [5.70, 8.97]	<0.001
**ΔCt *CELF4* (median [IQR])**	15.90 [10.87, 17.61]	16.10 [11.05, 17.76]	9.84 [7.83, 15.99]	6.35 [4.97, 8.59]	<0.001

CA125, carbohydrate antigen 125; BMI, body mass index; BE, benign endometrium; EH, hyperplasia without atypia; AH, atypical hyperplasia; EC, endometrial cancer; IQR, interquartile range.

According to the FIGO classification, of the 39 women with EC, 32 (82.1%) were in stage I, 3 (7.7%) in stage II, 3 (7.7%) in stage III, and 1 (2.5%) in stage IV. Regarding tumor grading, 7 (17.9%) were G1, 24 (61.5%) were G2, and 8 (20.5%) were G3 (**Supplementary S1**). There were 37 (97.4%) type I and 2 (2.6%) type II EC patients. No significant difference in DNA methylation (ΔCt *CDO1* value or ΔCt *CELF4* value) was found between different FIGO classifications (**Figure 2D**) and tumor grade (p > 0.05, **Figure 2E**). The methylation positivity rate was higher in postmenopausal women than in premenopausal women in all different pathological subgroups (**Figure 2F**).

### Analysis of clinical factors related to endometrial malignant lesions

A total of 607 patients were divided into the endometrium malignant group (AH and EC, 53 patients) and the endometrium benign group (BE and EH, 554 patients). As shown in **Table 2**, women in the malignant group were aged 55.0 [52.0–59.0] years, and those in the benign group were aged 46.0 [40.0–51.0] years. Age was significantly different between the two groups (p < 0.05). In addition to age, menopausal state, endometrial thickness ≥ 11 mm (premenopausal), endometrial thickness ≥ 5 mm (postmenopausal), BMI ≥ 25 kg/m^2^, ΔCt *CDO1* ≤ 8.4, ΔCt *CELF4* ≤ 8.8, *CDO1^m^*/*CELF4^m^*, polyp, and hypertension were significantly different in the malignant group and the benign group (all p < 0.05). Multifactorial analysis revealed that endometrial thickness ≥ 11 mm (premenopausal) and endometrial thickness ≥ 5 mm (postmenopausal), ΔCt *CDO1* ≤ 8.4, and ΔCt *CELF4* ≤ 8.8 were risk factors for the development of endometrial malignant lesions, with odds ratios (ORs) of 5.03 (95%CI: 1.83–13.82) (p < 0.05) and 6.92 (95%CI: 1.10–43.44) (p = 0.04) in **Table 3**.

**Table 2:** Univariate analysis of clinical indicators associated with endometrial malignant lesions.

		Overall (n = 607)	BE/EH (n = 554)	AH/EC (n = 53)	Z/χ²	p
**AGE (median [IQR])**		47.00 [40.00, 53.00]	46.00 [40.00, 51.00]	55.00 [52.00, 59.00]	1.097	<0.001
**Menopausal state (%)**	Premenopausal	465 (76.61)	447 (80.69)	18 (33.96)	1.072	<0.001
	Postmenopausal	142 (23.39)	107 (19.31)	35 (66.04)		
**Endometrial thickness status (%)**	Normal	362 (60.33)	354 (64.48)	8 (15.69)	1.148	<0.001
	Abnormal	238 (39.67)	195 (35.52)	43 (84.31)		
**CA125 (%)**	<35	269 (77.30)	224 (75.68)	45 (86.54)	0.28	0.106
	≥35	79 (22.70)	72 (24.32)	7 (13.46)		
**Abnormal bleeding (%)**	No	205 (33.77)	197 (35.56)	8 (15.09)	0.484	0.002
	Yes	402 (66.23)	357 (64.44)	45 (84.91)		
**BMI (%)**	<25	326 (53.71)	305 (55.05)	21 (39.62)	0.313	0.043
	≥25	281 (46.29)	249 (44.95)	32 (60.38)		
**ΔCt *CDO1* (%)**	>8.4	532 (87.64)	514 (92.78)	18 (33.96)	1.541	<0.001
	≤8.4	75 (12.36)	40 (7.22)	35 (66.04)		
**ΔCt *CELF4* (%)**	>8.8	517 (85.17)	501 (90.43)	16 (30.19)	1.563	<0.001
	≤8.8	90 (14.83)	53 (9.57)	37 (69.81)		
***CDO1^m^*/*CELF4^m^* (%)**	Negative	488 (80.40)	480 (86.64)	8 (15.09)	2.049	<0.001
	Positive	119 (19.60)	74 (13.36)	45 (84.91)		
**Adenomyosis (%)**	No	539 (88.80)	488 (88.09)	51 (96.23)	0.306	0.106
	Yes	68 (11.20)	66 (11.91)	2 (3.77)		
**Leiomyoma (%)**	No	404 (66.56)	363 (65.52)	41 (77.36)	0.264	0.094
	Yes	203 (33.44)	191 (34.48)	12 (22.64)		
**Polyp (%)**	No	412 (67.87)	361 (65.16)	51 (96.23)	0.856	<0.001
	Yes	195 (32.13)	193 (34.84)	2 (3.77)		
**Hypertension (%)**	No	249 (72.38)	225 (76.27)	24 (48.98)	0.588	<0.001
	Yes	95 (27.62)	70 (23.73)	25 (51.02)		
**Diabetes (%)**	No	317 (92.15)	275 (93.22)	42 (85.71)	0.246	0.084
	Yes	27 (7.85)	20 (6.78)	7 (14.29)		

CA125, carbohydrate antigen 125; BMI, body mass index; CDO1^m^/CELF4^m^: ΔCt CDO1 ≤ 8.4 or ΔCt CELF4 ≤ 8.8; BE, benign endometrium; EH, hyperplasia without atypia; AH, atypical hyperplasia; EC, endometrial cancer; IQR, interquartile range.

**Table 3:** Multifactorial analysis of clinical indicators related to endometrial malignant lesions.

	Estimate	Std. error	Z value	p-Value	ORs (95%CI)
**Age**	0.02	0.03	0.64	0.52	1.02 (0.96–1.09)
**Menopausal state**	0.34	0.68	0.50	0.61	1.41 (0.37–5.37)
**Endometrial thickness status**	1.61	0.52	3.13	**<0.001**	5.03 (1.83–13.82)
**Abnormal bleeding**	0.91	0.55	1.65	0.10	2.49 (0.84–7.35)
**BMI ≥ 25**	0.24	0.47	0.51	0.61	1.27 (0.50–3.22)
**ΔCt *CDO1* ≤ 8.4**	0.46	0.70	0.66	0.51	1.59 (0.40–6.30)
**ΔCt *CELF4* ≤ 8.8**	0.81	0.66	1.23	0.22	2.26 (0.62–8.22)
***CDO1^m^*/*CELF4* ^m^**	1.93	0.94	2.06	**0.04**	6.92 (1.10–43.44)
**Polyp**	−0.96	0.82	−1.17	0.24	0.38 (0.08–1.92)
**Hypertension**	0.63	0.49	1.28	0.20	1.88 (0.72–4.91)

Endometrial thickness status: endometrial thickness ≥ 11 mm in premenopausal groups or endometrial thickness ≥ 5 mm in postmenopausal groups.

BMI, body mass index; CDO1^m^/CELF4^m^, ΔCt CDO1 ≤ 8.4 or ΔCt CELF4 ≤ 8.8; ORs, odds ratios.

The bold values mean values with significant statistical meaning.

### Clinical performance of different tests for endometrial malignant lesion detection

**Table 4** reports the risk factors for the development of endometrial malignant lesions as the diagnostic performance test when applied to the study. In the study, the best AUC was 0.86 (0.81–0.91), with a sensitivity of 84.9% (75.3%–94.5%) and a specificity of 86.6% (83.8%–89.5%) by *CDO1^m^*/*CELF4^m^* for test endometrial malignant lesion patients. ETS/*CDO1m*/*CELF4m* test had the best sensitivity of 92.5% (85.3%–99.6%) and the best specificity of 94.9% (93.1%–96.8%) by ETS&(*CDO1^m^*/*CELF4^m^*) in all patients no matter the menopausal status. Compared with the premenopausal and postmenopausal groups, the AUC was 0.78 (0.66–0.89) by *CDO1^m^*/*CELF4^m^*, ET ≥ 11 mm/*CDO1^m^*/*CELF4^m^* test had the best sensitivity 83.8% (66.1%–100.0%), and ET ≥ 11 mm&(*CDO1^m^*/*CELF4^m^*) had the best specificity 96.4% (94.7%–98.1%) in the premenopausal group. ET ≥ 5 mm/*CDO1^m^*/*CELF4^m^* test had the best sensitivity of 97.1% (91.6%–100.0%), and ET ≥ 5 mm&(*CDO1^m^*/*CELF4^m^*) had the best AUC of 0.90 (0.85–0.96) and the best specificity of 88.8% (82.8%–94.8%) in the postmenopausal group.

**Table 4:** Diagnostic performance of single test and combined test.

	Sensitivity (95%CI)	Specificity (95%CI)	PPV (95%CI)	NPV (95%CI)	AUC (95%CI)
AH/EC (all, n = 53)					
**ETS (+)**	84.3 (74.3–94.3)	64.5 (60.5–68.5)	18.1 (26.4–23)	97.8 (96.3–99.3)	0.74 (0.69–0.80)
***CDO1^m^*/*CELF4* ^m^**	84.9 (75.3–94.5)	86.6 (83.8–89.5)	37.8 (58.2–46.5)	98.4 (97.2–99.5)	0.86 (0.81–0.91)
**ETS/*CDO1^m^*/*CELF4^m^***	92.5 (85.3–99.6)	56.5 (52.4–60.6)	16.9 (25.2–21.2)	98.7 (97.5–100)	0.75 (0.70–0.79)
**ETS&(*CDO1^m^*/*CELF4* ^m^)**	73.6 (61.7–85.5)	94.9 (93.1–96.8)	58.2 (46.4–70)	97.4 (96.1–98.7)	0.84 (0.78–0.90)
AH/EC (premenopausal, n = 19)					
**ET ≥ 11 mm**	58.8 (35.4–82.2)	69.9 (65.6–74.2)	7 (5.6–11.2)	97.8 (96.2–99.4)	0.64 (0.52–0.77)
***CDO1^m^*/*CELF4* ^m^**	66.7 (44.9–88.4)	88.4 (85.4–91.3)	18.8 (18.4–28.3)	98.5 (97.3–99.7)	0.78 (0.66–0.89)
**ETS/*CDO1^m^*/*CELF4* ^m^**	83.3 (66.1–100)	62.2 (57.7–66.7)	8.2 (8.4–12.1)	98.9 (97.7–100)	0.73 (0.64–0.82)
**ETS&(*CDO1^m^*/*CELF4* ^m^)**	38.9 (16.4–61.4)	96.4 (94.7–98.1)	30.4 (11.6–49.2)	97.5 (96.1–99)	0.68 (0.56–0.79)
AH/EC (postmenopausal, n = 34)					
**ET ≥ 5m m**	97.1 (91.4–100)	42.1 (32.7–51.4)	34.7 (50.4–44.3)	97.8 (93.6–100)	0.70 (0.64–0.75)
***CDO1^m^*/*CELF4* ^m^**	94.3 (86.6–100)	79.4 (71.8–87.1)	60 (94.2–72.9)	97.7 (94.6–100)	0.87 (0.81–0.92)
**ETS/*CDO1^m^*/*CELF4* ^m^**	97.1 (91.6–100)	32.7 (23.8–41.6)	32.1 (46.4–41)	97.2 (91.9–100)	0.65 (0.60–0.70)
**ETS&(*CDO1^m^*/*CELF4* ^m^)**	91.4 (82.2–100)	88.8 (82.8–94.8)	72.7 (59.6–85.9)	96.9 (93.5–100)	0.90 (0.85–0.96)

AUC, area under the receiver operating characteristic curve; PPV, the positive predictive value; NPV, the negative predictive value; ETS (+), the endometrial thickness status, endometrial thickness ≥ 11 mm in premenopausal groups or endometrial thickness ≥ 5 mm in postmenopausal groups; ET, endometrial thickness; CDO1^m^/CELF4^m^, ΔCt CDO1 ≤ 8.4 or ΔCt CELF4 ≤ 8.8; ETS/CDO1^m^/CELF4^m^, ETS (+) or CDO1^m^/CELF4^m^; ETS&(CDO1^m^/CELF4^m^), ETS (+) and CDO1^m^/CELF4^m^.

### Comparison of the accuracy of DNA methylation and transvaginal ultrasound

**Figure 3:**
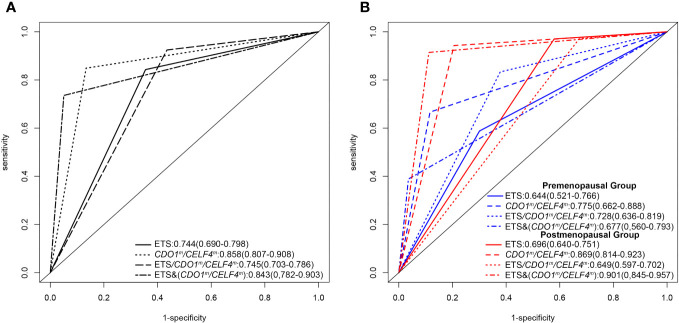
Receiver operating characteristic (ROC) curve analysis of difference testing. **(A)** ROC for endometrial malignant lesions of difference testing. **(B)** ROC for endometrial malignant lesions of difference testing in different menopausal states. ETS, the endometrial thickness status, endometrial thickness ≥ 11 mm in premenopausal groups or endometrial thickness ≥ 5 mm in postmenopausal groups; *CDO1^m^*/*CELF4* ^m^: ΔCt *CDO1* ≤ 8.4 or ΔCt *CELF4* ≤ 8.8; ETS/*CDO1^m^*/*CELF4* ^m^: ETS (+) or *CDO1^m^*/*CELF4* ^m^; ETS&(*CDO1^m^*/*CELF4* ^m^): ETS (+) and *CDO1^m^*/*CELF4* ^m^.

Validation of the diagnostic accuracy of the *CDO1^m^*/*CELF4^m^* test in 607 exfoliated cervical cell samples in the detection of EC and AH (**Figure 3A**), with endometrial malignant lesions as the outcome, resulted in an AUC of 0.86 (0.81–0.91), comparing endometrial benign groups. Stratification by menopausal status premenopausal and postmenopausal led to AUCs of 0.78 (0.66–0.89) and 0.87 (0.81–0.92), respectively (**Figure 3B**). DNA methylation combined with TVS could not further improve diagnostic accuracy in the premenopausal state [*CDO1^m^*/*CELF4* ^m^: 0.78 (0.66–0.89) *vs.* ETS/*CDO1^m^*/*CELF4^m^*: 0.73 (0.64–0.82)] but can further improve diagnostic accuracy in the postmenopausal state (*CDO1m*/*CELF4*m: 0.87 (0.81–0.92) *vs.* ETS&(*CDO1^m^*/*CELF4^m^*): 0.90 (0.85–0.96)). The diagnostic accuracy of the *CDO1^m^*/*CELF4^m^* in the detection of endometrial malignant lesions has no significant difference in premenopausal and postmenopausal states (p = 0.1453).

## Discussion

In this study, a non-invasive liquid biopsy protocol with cervical exfoliative cytology was used to detect endometrium malignant lesions by targeted genetic testing in women with indications for hysteroscopy. We proved that *CDO1* or *CELF4* methylation tests have relatively high sensitivity, specificity, and AUC in cervical cytological detection for EC and AH. When these two candidate genes are tested in combination, they perform better in clinical applications due to higher negative predictive value and AUC.

The expert consensus on endometrial cancer screening and early diagnosis identified anovulatory abnormal uterine bleeding as a high-risk factor for endometrial cancer [16]. For screening with symptoms of vaginal bleeding or endometrial thickening on ultrasound, progestin therapy can be tried first, and hysteroscopic detection is recommended for those who fail to be treated. Pathological examination after diagnostic curettage is important for the diagnosis and guidance of treatment of abnormal uterine bleeding [17]. Although diagnostic curettage is short, it can cause pain in patients, thus making the procedure more difficult for the surgeon and increasing the risk of surgery for the patient. In this study, women with abnormal uterine bleeding, vaginal drainage, and imaging suggestive of abnormalities during routine gynecological examination with indications for hysteroscopic detection were selected for enrollment, and the advantages of TVS versus DNA methylation testing in the screening and early diagnosis of endometrial malignant lesions were compared and analyzed.

The selection of cervical exfoliated cells for DNA methylation testing for the diagnosis of endometrial cancer is an important innovation in this study. Studies have been conducted on the use of exfoliated cells for cytopathological analysis, target gene detection and genetic syndrome confirmation, exosome analysis, microsatellite instability (MSI) testing, and multi-omics testing (50–55). Recent foreign studies suggest that cervical exfoliative cell DNA methylation testing may be a patient-friendly tool for screening and triaging women with endometrial atypical hyperplasia or endometrial cancer-like conditions or at risk for endometrial malignancy, and because it is suitable for self-collected samples, it may be an appropriate tool for managing women with abnormal uterine bleeding, and future implementation of such testing protocols in preventive screening and early detection settings is being considered (56). However, no studies related to epigenetic analysis of exfoliated cytology have been conducted in China, and there is no reliable screening protocol for clinical application. The use of cervical exfoliated cells for endometrial cancer screening and diagnosis has the outstanding advantages of being completely non-invasive, easy to obtain, and highly accurate (57), having adequate cell volume, and being highly consistent with histological findings [15]. The association of epigenetic alterations in *CDO1* and *CELF4*, the target genes selected in this study, with endometrial malignant lesions has been supported in the literature (33, 58). In histological specimens from EC and AH patients, *CDO1* and *ZNF454* are hypermethylated compared with benign and normal endometrium (p < 0.001). Also, in a total of 120 cytological specimens, the AUC of the diagnostic test and the methylation biomarker panel was 0.931, with a sensitivity of 9.91% and a specificity of 86.84% (39). In addition, another panel of three genes, *BHLHE22*, *CDO1*, and *CELF4*, including any two of the three hypermethylated genes, reached a sensitivity of 91.8% and a specificity of 95.5%. Different from previous studies, this study confirms the effectiveness of *CDO1^m^*/*CELF4^m^* in screening and triage of women with endometrial biopsy indications, and they can effectively diagnose and shunt women with symptoms or risks of AH and EC. In our research, the AUC was 0.86 (0.81–0.91), with a sensitivity of 84.9% (75.3%–94.5%) and a specificity of 86.6% (83.8%–89.5%) in *CDO1^m^/CELF4^m^* testing for endometrial malignant lesion patients.

Traditionally, ET is a very important indicator in endometrial cancer screening. TVS has become the preferred screening method for clinical diagnosis of endometrial disease with the advantages of being non-invasive, economical, and easy to perform (59). TVS is highly sensitive for screening endometrial cancer but has low specificity and often results in unnecessary invasive operations. The 2018 Cancer Report published by the International Federation of Obstetrics and Gynecology in October 2018 on endometrial cancer screening states that TVS combined with endometrial diagnostic scraping biopsy has a negative predictive value of 96%, but some patients do not want to undergo the invasive operation of segmental scraping (60, 61). In this study, DNA methylation assay (*CDO1^m^*/*CELF4^m^*) had a high sensitivity of 84.9% (75.3%–94.5%) and a specificity of 86.6% (83.8%–89.5%), and the combination of ETS/*CDO1^m^*/*CELF4^m^* could further improve the sensitivity 92.5% (85.3%–99.6%) of endometrial malignant lesion detection. Furthermore, the combined ETS&(*CDO1^m^*/*CELF4^m^*) can achieve a higher level of specificity of 94.9% (93.1%–96.8%). By combining with TVS in different ways, the specificity can be further enhanced, and the possibility of invasive manipulation is reduced. Whether this situation can be repeated and improved remains to be confirmed by large cohort studies. In this study, DNA methylation was an independent predictor of endometrial malignant lesions. DNA methylation had high sensitivity of 97.1% (91.6%–100%) in the postmenopausal subgroup, detecting more patients with endometrial malignant lesions, while in the non-menopausal group, it had higher specificity of 88.4% (85.4%–91.3%), providing better triage for patients with abnormal bleeding and endometrial thickening, avoiding overly invasive consultations in women of reproductive age, and serving to protect female fertility.

Previous studies have shown a correlation between serum glycoantigen CA125 and the pathological stage of endometrial cancer, and according to the trend of CA125, patients with endometrial cancer can be effectively screened to some extent. However, the rate of misdiagnosis and leakage is also relatively high, and it needs to be combined with other detection methods to improve diagnostic efficacy (62). In this study, there was no statistically significant difference in CA125 levels in the benign endometrial group, hyperplasia without atypia group, atypical hyperplasia group, and endometrial cancer group (p = 0.444). It may be related to the elevated CA125 levels in patients with uterine smooth muscle tumors, adenomyoma (or combined adenomyosis) in the benign endometrial group, and hyperplasia without the atypia group. BMI was not significantly different in the benign endometrial group, hyperplasia without atypia group, atypical hyperplasia group, and endometrial cancer group (p = 0.1). When patients were divided into the endometrium malignant group (AH and EC) and the endometrium benign group (BE and EH), the proportion of BMI ≥ 25 kg/m^2^ in the endometrium malignant group was 32 (60.38%), which was significantly higher than in the endometrium benign group at 249 (44.95%), χ^2 = ^0.313 and p = 0.043. A recent meta-analysis of 30 prospective studies reported that each 5 kg/m^2^ increase in BMI was associated with a 54% (95%CI: 47%–61%) higher risk of endometrial cancer (63, 64). The occurrence of endometrial polyps in the endometrium malignant group at 2 (3.77%) was significantly lower than in the endometrium benign group at 193 (34.84%), χ^2 = ^0.856 and p < 0.05. This is consistent with previous reports in the literature that most endometrial polyps are benign overgrowths of endometrial mucosa and that spontaneous regression can occur (65, 66).

In this study, cytological methylation testing was used for the detection of malignant endometrial lesions, which can reduce invasive testing of the uterus in women of reproductive age and provide a positive impetus to protect female fertility. The enrollment criteria for this study were the women with indications for endometrial biopsy, the vast majority of which were benign lesions, but malignant lesions were also easily overlooked. Methylation testing is objective, non-invasive, and accurate and can be accurately diagnosed for graded treatment in hospitals where pathologists have limited diagnostic skills. This study also has one limitation: this study selected patients screened in a single hospital gynecology department within 1 year, and the sample size of cancer (n = 39) and atypical hyperplasia patients (n = 14) included in the cohort study is small, with certain geographical limitations. There is an urgent need for future promotion in a large cohort and other problems.

## Conclusions

We demonstrated the effectiveness of *CDO1^m^*/*CELF4^m^* in screening and triaging women with endometrial biopsy indications, and they can be effective in diagnosing and triaging women with symptoms or at risk for AH and EC. The *CDO1* and *CELF4* dual-gene methylation test provides a simple and highly accurate non-invasive management method for women with abnormal uterine bleeding, which reduces most invasive operations, reduces the psychological burden on patients, and improves patient compliance during the testing process.

## Data availability statement

All data generated or analyzed during this study are included in this published article.

## Ethics statement

The study was approved by the Ethics Committee of the Cangzhou Central Hospital (No. 2023-144-02(z)), and it was conducted in accordance with the Declaration of Helsinki. Patients with indications for endometrial biopsy were enrolled and provided signed informed consent according to the Standards for Reporting of Diagnostic Accuracy Studies (STARD) in clinics.

## Author contributions

BQ: Conceptualization, Methodology, Writing – original draft, Writing – review & editing. YS: Formal analysis, Methodology, Writing – review & editing. YHL: Data curation, Methodology, Writing – review & editing. PH: Data curation, Formal analysis, Writing – review & editing. YM: Investigation, Methodology, Writing – review & editing. WG: Investigation, Methodology, Writing – review & editing. SML: Data curation, Software, Writing – review & editing. XZ: Conceptualization, Data curation, Writing – review & editing. XJ: Conceptualization, Data curation, Writing – original draft. YLL: Conceptualization, Writing – review & editing. PL: Funding acquisition, Writing – review & editing. SKL: Conceptualization, Data curation, Formal analysis, Methodology, Resources, Writing – original draft, Writing – review & editing.
